# Differential effects of allopregnanolone and diazepam on social behavior through modulation of neural oscillation dynamics in basolateral amygdala and medial prefrontal cortex

**DOI:** 10.3389/fncel.2024.1404603

**Published:** 2024-06-05

**Authors:** Yosuke Yawata, Ryoichi Tashima, Hiroyuki Aritomi, Shinji Shimada, Tsukasa Onodera, Teruhiko Taishi, Keiko Takasu, Koichi Ogawa

**Affiliations:** ^1^Laboratory for Drug Discovery and Disease Research, Shionogi Pharmaceutical Research Center, Shionogi & Co., Ltd., Osaka, Japan; ^2^Shionogi TechnoAdvance Research, Osaka, Japan

**Keywords:** neuroactive steroid, benzodiazepine, extrasynaptic GABA_A_ receptor, social defeat stress model, antidepressant-like effect, theta activity, basolateral amygdala

## Abstract

Effective treatments for major depressive disorder (MDD) have long been needed. One hypothesis for the mechanism of depression involves a decrease in neuroactive steroids such as allopregnanolone, an endogenous positive allosteric modulator of the γ-aminobutyric acid–gated chloride channel (GABA_A_) receptor. In our previous study, we discovered that allopregnanolone, not diazepam, exhibited antidepressant-like effects in the social interaction test (SIT) of social defeat stress (SDS) model mice. However, the dynamics of neuronal activity underlying the antidepressant-like effect remain unknown. In the current study, we conducted local field potentials (LFPs) recordings from the basolateral amygdala (BLA) and the medial prefrontal cortex (mPFC) during the SIT to elucidate the relationship between the antidepressant-like effect and neuronal oscillation. We discovered that allopregnanolone has antidepressant-like effects in the SIT of SDS model mice by decreasing intervals of repetitive social interaction (inter-event intervals), resulting in increase of total social interaction time. We also found that theta and beta oscillation increased in BLA at the onset of social interaction following administration of allopregnanolone, which differed from the effects of diazepam. Theta and beta power in BLA within the social interaction zone exhibited a positive correlation with interaction time. This increase of theta and beta power was negatively correlated with inter-event intervals. Regarding theta-band coordinated activity between the BLA and mPFC, theta power correlation decreased at the onset of social interaction with the administration of allopregnanolone. These findings suggest that theta activity in BLA following social interaction and the reduced theta-band coordinated activity between the BLA and mPFC are implicated in social interaction, which is one of the antidepressant behaviors. These differences in neural activity could elucidate the distinctive mechanism underlying antidepressant-like effects of neuroactive steroids, as opposed to benzodiazepines.

## Introduction

The need for effective treatments for major depressive disorder (MDD) has become a pressing medical requirement. MDD is characterized by an episode of core depressive symptoms lasting at least 2 weeks, including pervasive low mood or loss of interest or pleasure in typically enjoyable activities (American Psychiatric Association, 2013). While various treatments for depression exist, there remains a crucial demand for novel and more efficacious approaches to alleviate the burden of this debilitating disorder. In this pursuit, increasing attention has been drawn to the potential therapeutic role of neuroactive steroids. Recently, new antidepressant drugs, brexanolone (allopregnanolone) and zuranolone (SAGE-217), received approval from Food and Drug Administration for the treatment of postpartum depression, and zuranolone continues to be studied in Japan for use in MDD. These drugs are neuroactive steroids that act on both synaptic and extrasynaptic GABA_A_ receptors (Edinoff et al., 2021; Ali et al., 2023; Kato et al., 2023). Previous studies have reported that allopregnanolone, an endogenous neuroactive steroid, in plasma or brain is decreased in MDD patients and there is a negative correlation between the concentration of allopregnanolone and the severity of depressive symptoms (Uzunova et al., 1998; Agis-Balboa et al., 2014). Moreover, increased plasma concentrations of allopregnanolone with antidepressant treatment are associated with relief of depressive symptoms (Romeo et al., 1998). Conversely, benzodiazepines do not receive approval for the treatment of patients with MDD (Lim et al., 2020), while they also act as positive allosteric modulators of the GABA_A_ receptor. This difference in efficacies in patients with MDD could be explained by the different molecular mechanisms. That is, neuroactive steroids potentiate both synaptic GABA_A_ receptor, which contains the α, β, and γ subunits, and extrasynaptic one, which contains the α, β, and δ subunits. In contrast, benzodiazepines only potentiate γ subunit-containing GABA_A_ receptor, which are primarily synaptic. Therefore, δ subunit-containing extrasynaptic GABA_A_ receptor could largely contribute to difference in the efficacies. This concept has been proposed in recent studies (Meltzer-Brody and Kanes, 2020; Reddy et al., 2023).

Previous studies examined the effects of neuroactive steroids on depressive symptoms and neural activity. These studies demonstrated that theta oscillation (6–12 Hz) in basolateral amygdala (BLA) is regulated by extrasynaptic GABA_A_ receptors on interneurons and can contribute to the antidepressant-like effects in chronic unpredictable stress model mice (Antonoudiou et al., 2022; Walton et al., 2023). Notably, they also showed a correlation between resting-state BLA theta power of mice treated with neuroactive steroid and a reduction of immobility time in the tail suspension test (TST). Recently, we reported that allopregnanolone has antidepressant-like effects in social defeat stress (SDS) model mice, as evidenced by a decrease in immobility time in the TST and an increase in social interaction time in the social interaction test (SIT), respectively (Takasu et al., 2024). We also demonstrated that allopregnanolone increased theta and beta (15–30 Hz) activities in the BLA and medial prefrontal cortex (mPFC), whereas diazepam only increased beta activity in the resting state LFPs of mice. However, these studies have been limited in scope, primarily examining neural activities during resting state in the home cage. Specifically, the precise temporal dynamics of neural activities during the social behavior associated with the manifestation of antidepressant-like effects are still unknown.

To address this question directly, we recorded the LFPs in BLA and mPFC from SDS model mice while conducting the SIT, enabling us to associate neural activity with specific behavior such as social interaction. We investigated the oscillation in BLA and mPFC, as well as the coordinated activity between these regions associated with antidepressant-like effects elicited by neuroactive steroids, which differs from the effects of benzodiazepines.

## Materials and methods

### Animals

Experiments were performed using C57BL/6 J Jcl mice and Crl:CD1 (ICR) retired mice. ICR mice (male) were purchased from Jackson Laboratory. C57BL/6 J Jcl mice (male) were purchased from CLEA Japan, Inc. and were used as wild-type controls. The body weights of C57BL/6 J Jcl mice were 20–30 g. Mice aged 2–4 months were used. The mice were housed under controlled temperature and humidity with a 12/12-h light/dark cycle (light from 8:00 to 20:00). Less than three mice were housed in a cage (W 235 mm, D 353 mm, H 160 mm) with bedding paper chips (SLC Japan, Inc.) and Nesting Sheets™ (Bio-Serv, United States) under environmental enrichment. Mice were allowed *ad libitum* access to food (CE-2, CLEA Japan, Inc.) and clean water (filtered at 5 mm from Toyonaka City, Japan) under SPF conditions. All procedures were approved by the Animal Care and Use Committee of Shionogi Research Laboratories, Osaka, Japan. Electrophysiological assessments were performed according to the Association for Assessment and Accreditation of Laboratory Animal Care (AAALAC) International guidelines.

### Drugs

Allopregnanolone was purchased from Toronto Research Chemicals (Canada). Diazepam and was purchased from Fujifilm (Japan). Hydroxypropyl-β-cyclodextrin was purchased from Tokyo Chemical Industry (Japan). Allopregnanolone and diazepam were dissolved in 15% hydroxypropyl-β-cyclodextrin in distilled water. Allopregnanolone and diazepam were administered intraperitoneally.

### Preparation of SDS model

**Figure 1:**
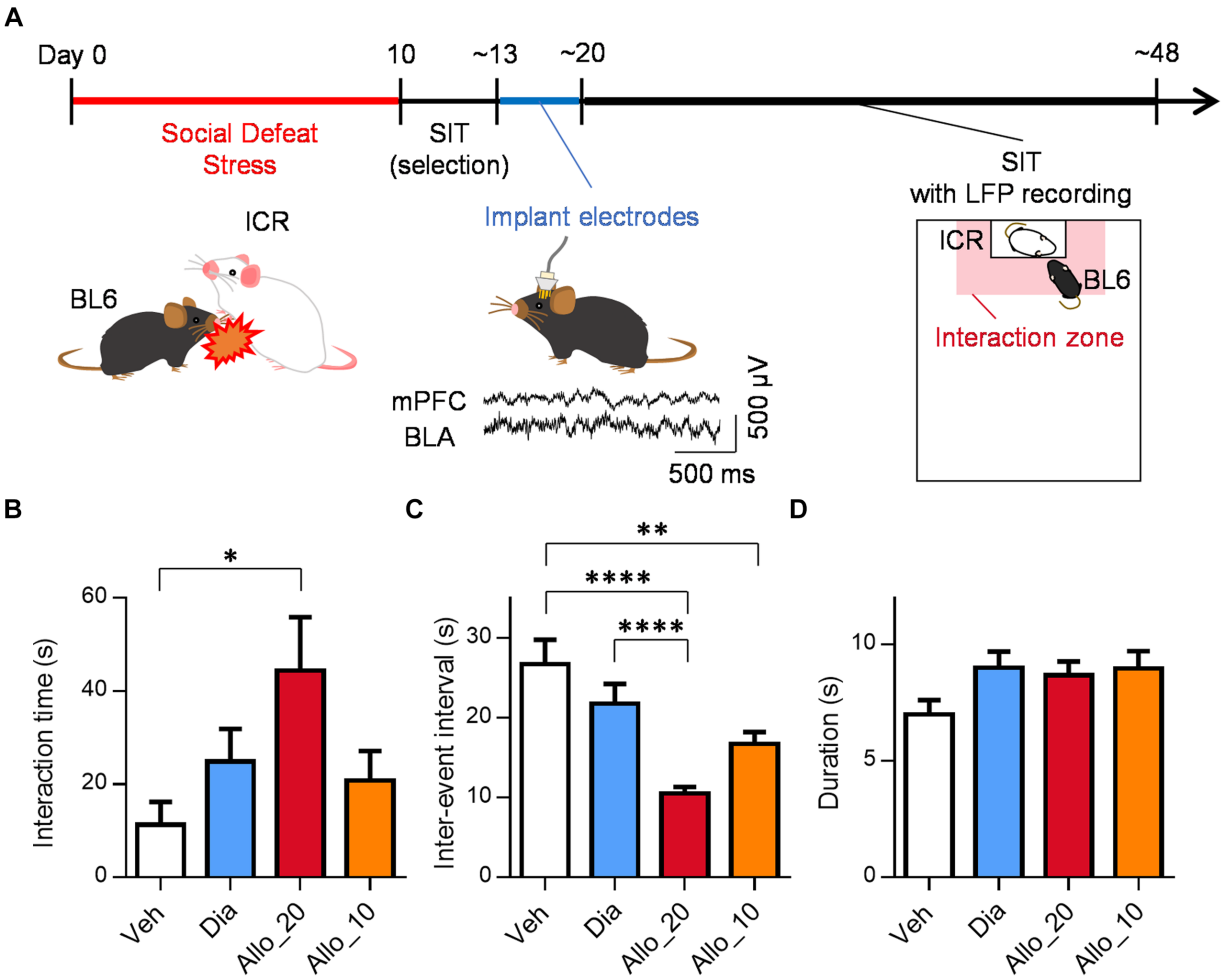
Allopregnanolone, but not diazepam, elicits antidepressant-like effects. **(A)** Behavioral paradigm of antidepressant assessment in the social interaction test (SIT) of the social defeat stress (SDS) model. A C57BL/6 J Jcl (BL6) mouse was defeated by a larger ICR mouse for 10 days. SIT was conducted to select depression-like mice, which were implanted LFP electrodes in the medial prefrontal cortex (mPFC) and basolateral amygdala (BLA). The center bottom traces are representative LFPs in the mPFC and BLA. After 1 week recovery, the mice were administered drugs (vehicle, diazepam, or allopregnanolone), followed by the SIT with LFP recordings. Time in the interaction zone (shaded in red) and frequency of entering the interaction zone were measured. **(B)** Interaction time after administration of vehicle (Veh, white column), diazepam (Dia, blue column), allopregnanolone 20 mg/kg (Allo_20, red column) and allopregnanolone 10 mg/kg (Allo_10, orange column) for 2.5 min. Data are represented as the mean ± SEM. ^*^p = 0.020, *n* = 18, 18, 18, and 11 mice for Veh, Dia, Allo_20, and Allo_10, respectively, Tukey’s test. **(C)** Inter-event interval of social interaction event after administration of Veh, Dia, Allo_20, and Allo_10. Data are represented as the mean ± SEM. ^**^p = 0.0047, ^****^p < 0.0001, *n* = 203, 238, 353, and 204 events for Veh, Dia, Allo_20, and Allo_10, respectively, Tukey’s test. **(D)** Duration of social interaction event after administration of Veh, Dia, Allo_20, and Allo_10. Data are represented as the mean ± SEM. *n* = 214, 252, 366, and 210 events for Veh, Dia, Allo_20, and Allo_10, respectively, Tukey’s test.

A C57BL/6 J Jcl mouse was defeated by a larger stranger ICR mouse as previously described (Takasu et al., 2024). Briefly, each individual defeat lasts 10 min (Figure 1A). If the mice developed severe injuries or extreme weakness during the 10-day defeat procedure, they could be euthanized at the veterinarian’s discretion. After the 10-day defeat procedure, SIT was conducted to select the mice with depression-like behavior with a series of sequential 3 tests: (1) Each C57BL/6 J Jcl mouse was placed in an open field chamber (40 × 30 × 20 cm) with an empty a plastic box at one end, and the mouse was allowed to freely explore the chamber for 150 s. This session was defined as test 1. (2) After that day, the C57BL/6 J Jcl mouse was placed in an open field chamber where a novel male ICR mouse was enclosed in a plastic box at one end, and the mouse was allowed to freely explore the chamber for 150 s under video recording. This session was defined as test 2. (3) After that day, the same test as test 2 was conducted. This session was defined as test 3. In each test, the time that each mouse spent in the interaction zone (5 cm around the plastic box with or without the ICR mouse) was calculated by tracking the mass of the mouse with the EthoVision XT video-tracking system (Noldus Information Technology, Wageningen, The Netherlands). The times in tests 1, 2, and 3 were defined as direct SIT time 1 (dSIT1), direct SIT time 2 (dSIT2) and direct SIT time 3 (dSIT3), respectively. A mouse whose dSIT2 and dSIT3 were both lower than dSIT1 was regarded as a depression-like mouse. Depression-like mice were used for *in vivo* electrophysiological studies with local field potential (LFP) recordings.

### Social interaction test

In depression-like mice, the SIT was conducted to evaluate the efficacy of the test substance. Each depression-like C57BL/6 J Jcl mouse was placed in an open field chamber where a novel male ICR mouse was enclosed in a plastic box at one end, and the mouse was allowed to freely explore the chamber for 600 s while being recorded on video. The time that each mouse spent in the direct interaction zone (close to the plastic box with the ICR mouse) was calculated by tracking the center of mass of the mouse with an EthoVision XT video-tracking system (Figure 1A). SIT was conducted 30 min after administration of allopregnanolone (10 or 20 mg/kg), diazepam (2 mg/kg), or vehicle. Data are expressed as the time that each mouse spent in the direct interaction zone, the inter-event interval that the period between the offset of the previous interaction and the onset of the present interaction, and the duration of each interaction event. Multiple group comparisons were performed using Tukey’s test after one-way analysis of variance (ANOVA). A value of *p* < 0.05 was considered statistically significant.

### *In vivo* electrophysiological assessment

#### Surgery

Surgery was performed to implant electrodes for recording LFPs and electromyography (EMG) signals from mice. The detailed surgical procedures have been previously described in Yawata et al. (2023) and Takasu et al. (2024). Briefly, LFP electrode devices consisting of a core body and a custom-made electrical interface board accommodating 6–12 LFP channels, 2 EMG channels, and 1 ground/reference channel were assembled for LFP recordings before surgery. For the surgery, mice were anesthetized with 1%–2.5% isoflurane gas. After anesthesia, a midline incision was made from the area between the eyes to the incised neck area, and 2 stainless-steel EMG electrodes with a tip diameter of 147 μm (AS633; Cooner Wire Company) in which the PTFE coating at the tip (~5.0 mm) was peeled off were sutured to the dorsal neck muscles. Circular craniotomies with a diameter of ~1 mm were made using a high-speed drill above the mPFC (1.9 mm anterior and 0.2 mm right to the bregma), BLA (1.6 mm posterior and 3.0 mm right to the bregma), and cerebellum (5.8 mm posterior ±1.0 mm lateral to bregma) for the ground/reference. The dura was surgically removed. The tips of the BLA and mPFC electrodes were inserted 3.85 and 2.1 mm from the brain surface, respectively. Stainless-steel screws were implanted on the surface of the cerebellum (5.8 mm posterior and 1.0 mm right/left to the bregma) as ground/reference electrodes. All the wires and the electrode assembly were secured to the skull using dental cement.

#### *In vivo* electrophysiological recording

Approximately 1 week after surgery, each mouse was connected to recording equipment to record *in vivo* LFP signals. Recordings were conducted within a copper-shielded room to remove 60-Hz hum noise. LFPs were sampled at 2 kHz using Cerebus (Blackrock Microsystems, Salt Lake City, Utah, United States). The LFP data were filtered between 0.1 and 500 Hz. Home-cage recordings were conducted for 60 min. After a baseline recording for 30 min, the mice were administered vehicle, diazepam (2 mg/kg), or allopregnanolone (10 or 20 mg/kg) intraperitoneally, and recording continued for an additional 30 min. These drugs were administered sequentially on the same mice with an interval of at least 1 day. The order of administration was vehicle, diazepam, and then allopregnanolone (20 mg/kg). For the measurement of allopregnanolone (10 mg/kg), we administered vehicle, allopregnanolone (10 mg/kg), diazepam, and then allopregnanolone (20 mg/kg). After the home-cage recordings, the mouse was placed in the open field chamber and SIT was conducted with LFPs recording.

#### Histology

After all recordings and behavioral tests, mice implanted with LFP electrodes were euthanized with an overdose of isoflurane and perfused intracardially with PBS, followed by 4% paraformaldehyde in PBS. Brains were removed, postfixed overnight in 4% paraformaldehyde, and equilibrated in 30% sucrose in PBS overnight. Frozen coronal sections (50 μm) were cut using a cryostat (NX50, PHC Corporation) and mounted with DAPI-containing mounting medium (VECTASHIELD Vibrance Antifade Mounting Medium, Funakoshi). Fluorescence images were captured using an all-in-one microscope (BZ-X710; Keyence, Osaka, Japan). LFP recordings were excluded from the data analysis unless the electrode was in the BLA and the mPFC.

#### Data analysis

The analysis of LFP signals was conducted using NeuroExplorer (Plexon Inc., Dallas, Texas, United States). When collecting data from multiple electrodes in a mouse, we selected the data from the electrode within each region, the BLA and the mPFC, for analysis (Supplementary Figure S1). To compute the time-frequency representation of LFP power, the Fourier transform was applied to LFP signals, and the intensity of each frequency was calculated for 1-s bin. The LFP power values were normalized for each time point across the total power. The coherence between two electrodes was computed using a Coherence Analysis function by NeuroExplorer. The correlations of LFP power in each frequency band between two electrodes were calculated from the data which contains more than 10 s (i.e., more than 10 pairs of LFP power). For quantification of mPFC-BLA correlative activity (coherence and correlation), the data were obtained using representative mPFC-BLA electrode pairs, specifically selecting representative electrodes from these regions. To analyze LFP signals within the physiological range, periods in which the LFPs for 1-s bins exceeded 2 mV were excluded from the analysis. The missing values during the excluded periods were filled using linear interpolation. The definitions of each frequency band were based on previous studies (Antonoudiou et al., 2022) and set as follows: theta wave (6–12 Hz) and beta wave (15–30 Hz). The LFP power and the mPFC-BLA LFP power correlation at the onset of social interactions was calculated as the change from the baseline, the mean of −5~0 s of each interaction event. The LFP power of the mean of 3~5 s of each interaction was used for the comparison among vehicle, diazepam, and allopregnanolone. The interaction events which lasted for at least 5 s with an interval of at least 5 s from the previous event were used for the analysis of the LFP power and LFP power correlation. The coherence was similarly calculated, but for the baseline of the mean of −10~−5 s of each interaction event. The mPFC-BLA correlative activity of the mean of 0~5 s of each interaction was used for the comparison among vehicle, diazepam, and allopregnanolone. The interaction events which lasted for at least 5 s with an interval of at least 10 s from the previous event were used for the analysis of the coherence. The events with high variability in the baseline, defined as the difference between the maximum and minimum values exceeding the mean plus two standard deviations (mean + 2SD) of all events, were omitted from these analyses. The relationships between the mean inter-event interval of each mouse and LFP power difference between inside and outside the interaction zone throughout the entire experiment were calculated using the data from mice with three or more events. The relationships between the LFP signals in the interaction zone and the social interaction time were calculated in 150-s bins. Tukey’s test after one-way ANOVA was used for multiple comparison and Pearson correlation coefficient was calculated for the correlation analysis. *p* < 0.05 was taken to indicate statistical significance. All data are expressed as the means ± SEMs.

## Results

### Effects of allopregnanolone on social behavior, in contrast to diazepam

To investigate the potential antidepressant-like effects of neuroactive steroids, we assessed depression-like behavior using SDS model mice. This model exhibits various symptoms and pathologies associated with MDD and is commonly employed for evaluating the effects of drugs on depression-like behavior (Petković and Chaudhury, 2022; Takasu et al., 2024). In the SDS protocol, C57BL/6 J Jcl mice were subjected to defeat by larger ICR mice for a period of 10 days. Subsequently, we conducted the SIT to identify the depression-like mice that displayed reduced social interaction time and locomotor activity compared to their baseline levels before the SDS procedure (Supplementary Figures S2A,B; *p* < 0.0001 for dSIT1 vs. dSIT2 and dSIT3, *n* = 18 mice, Tukey’s test). For the depression-like mice, we surgically implanted a microdrive containing up to 12 LFP electrodes and an EMG electrode. These electrodes were inserted into the BLA and the mPFC. Following a one-week recovery period, we performed the SIT with simultaneous LFP recording to investigate neural activities associated with social behavior (Figure 1A). After all recordings, the location of the electrode tips was confirmed within their respective regions (Supplementary Figure S1).

We evaluated the antidepressant-like effects of neuroactive steroids allopregnanolone and the benzodiazepine diazepam. Allopregnanolone administered at a dose of 20 mg/kg (Allo_20) significantly increased social interaction time in the SIT of SDS mice compared to the vehicle control (Veh), whereas 2 mg/kg of diazepam (Dia) and 10 mg/kg of allopregnanolone (Allo_10) did not fully produce such effects (Figure 1B; *p* = 0.020, *n* = 18, 18, 18, 11 mice for Veh, Dia, Allo_20, and Allo_10, respectively, Tukey’s test). This result demonstrates the antidepressant-like effects of allopregnanolone are dose-dependent. Additionally, we examined individual interaction events. The inter-event interval, defined as the time between the end of one interaction and the beginning of the next, was shorter in Allo_20 condition compared to both Veh and Dia (Figure 1C; *p* < 0.0001, *n* = 203, 238, 353, 204 events for Veh, Dia, Allo_20, and Allo_10, respectively, Tukey’s test), and a similar result was observed in Allo_10 condition compared to Veh (Figure 1C; *p* = 0.0047, Tukey’s test). However, the duration of each interaction event did not differ among all conditions (Figure 1D; *n* = 214, 252, 366, 210 events for Veh, Dia, Allo_20, and Allo_10, respectively, Tukey’s test). It is possible that the increased social interaction time was due to restoration of locomotor activity following allopregnanolone administration: the distance traveled in the SIT was indeed greater in Allo_20 trials compared to Veh trials (Supplementary Figure S3A; *p* = 0.037, *n* = 18, 18, 18, 11mice for Veh, Dia, Allo_20, and Allo_10, respectively, Tukey’s test). Nevertheless, there were no significant correlations between the distance traveled and the social interaction time in each drug trial (Supplementary Figure S3B; 0.36, 0.23, 0.40, and-0.33, *p* = 0.14, 0.35, 0.099, and 0.33, *n* = 18, 18, 18, and 11 mice for Veh, Dia, Allo_20, and Allo_10, respectively, Tukey’s test), suggesting that the increase in social interaction time was not solely attributable to restoration of locomotor activity. These findings suggest that mice administered with allopregnanolone reduced the time until their next social interaction event, rather than increasing duration of individual interactions.

### Increase in theta and beta oscillation in BLA at the onset of social interaction with administration of allopregnanolone

**Figure 2:**
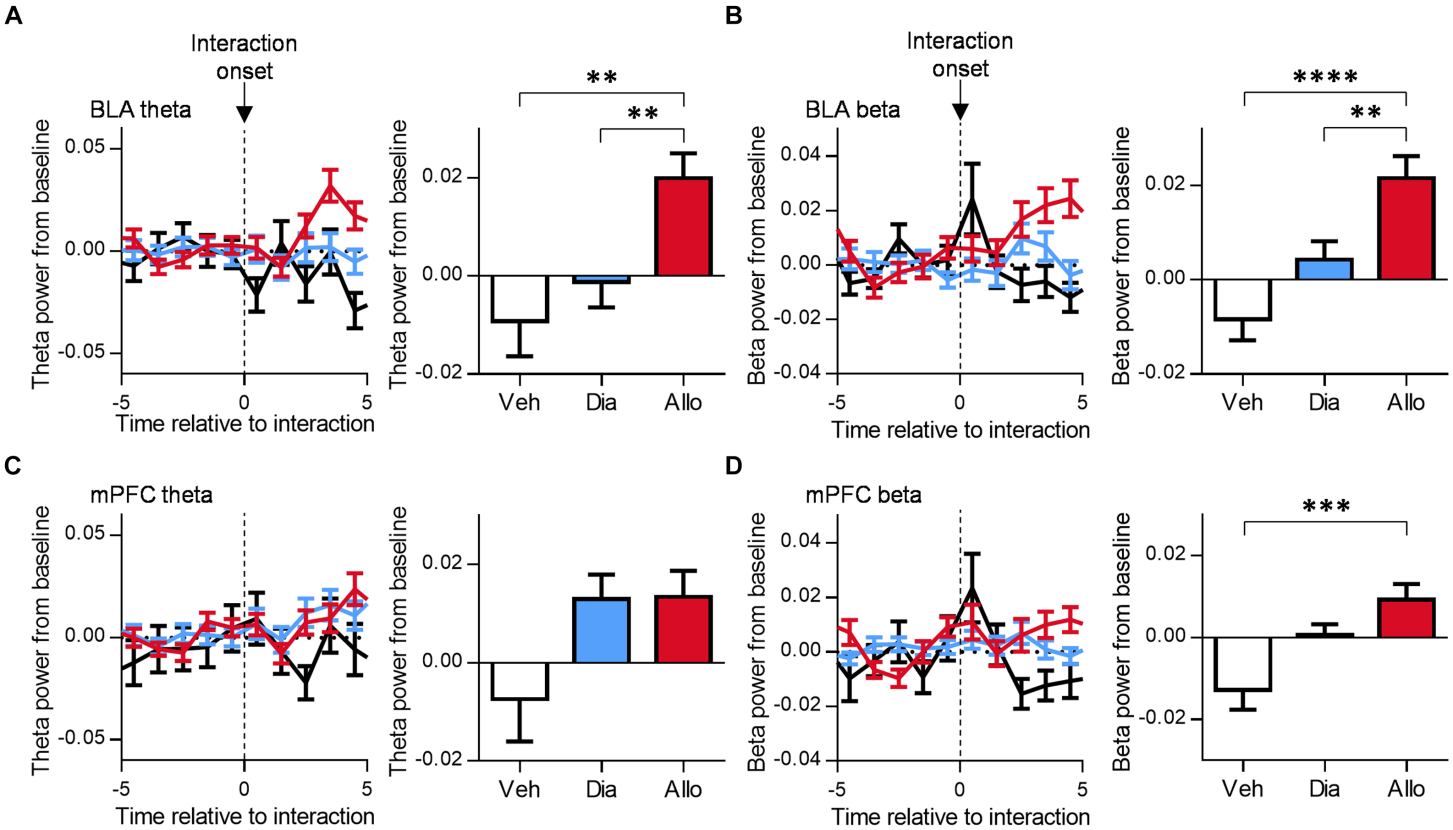
Allopregnanolone, but not diazepam, increases theta and beta oscillation in the BLA at the onset of social behavior. **(A)** Time course of theta power in the BLA around the onset of social interaction in administration of vehicle (Veh, black), diazepam (Dia, blue), and allopregnanolone (Allo, red). The power was normalized for each time point across the total power. Baseline was set at the mean of −5~0 s of each event. Right bar graph indicates the mean of 3~5 s after interaction. Data are represented as the mean ± SEM. ^**^p = 0.0029, 0.0095 for Veh vs. Allo, Dia vs. Allo, respectively, *n* = 56, 110, and 117 events for Veh, Dia, and Allo, respectively, Tukey’s test. **(B)** Same as **(A)**, but for beta power in the BLA. ^**^p = 0.0091, ^****^p < 0.0001, *n* = 63, 112, and 110 events for Veh, Dia, and Allo, respectively, Tukey’s test. **(C)** Same as **(A)**, but for the mPFC. *p* > 0.05, *n* = 59, 141, and 133 events for Veh, Dia, and Allo, respectively, Tukey’s test. **(D)** Same as **(B)**, but for the mPFC. ^***^p = 0.0006, *n* = 61, 146, 137 events for Veh, Dia, and Allo, respectively, Tukey’s test.

To investigate the neural activities correlated with antidepressant-like effects during social behavior, we focused on the LFPs recorded in BLA and mPFC, which have been previously implicated in depression in various studies (Drevets, 2000; Phillips et al., 2003; Deligiannidis et al., 2013; Chase et al., 2014; Deligiannidis et al., 2019). Furthermore, our previous study revealed an increase in theta (6–12 Hz) and beta (15–30 Hz) oscillations in BLA and mPFC following administration of allopregnanolone in mice within their home cages (Takasu et al., 2024), which was correlated with an increase in social interaction time. As a subsequent step, we examined the neural dynamics during social behavior by analyzing whether theta activity in BLA and mPFC increased at the onset of each social interaction event. The change in theta power from the baseline increased after the initiation of interaction event in the presence of allopregnanolone compared to vehicle and diazepam (Figure 2A; *p* = 0.0029 and 0.0095 for Veh vs. Allo, Dia and Allo, respectively, *n* = 56, 110, 117 events for Veh, Dia, and Allo, respectively, Tukey’s test.). Similarly, the beta power increased after the onset of events with allopregnanolone administration compared to vehicle and diazepam (Figure 2B; *p* < 0.0001 and *p* = 0.0091 for Veh vs. Allo, Dia and Allo, respectively, *n* = 63, 112, 110 events for Veh, Dia, and Allo, respectively, Tukey’s test.). Conversely, theta power in mPFC at the onset of social interaction did not differ among three drug conditions (Figure 2C; *p* > 0.05, *n* = 59, 141, 133 events for Veh, Dia, and Allo, respectively, Tukey’s test.), while beta power in mPFC increased with the administering of allopregnanolone (Figure 2D; *p* = 0.0006 for Veh vs. Allo, *n* = 61, 146, 137 events for Veh, Dia, and Allo, respectively, Tukey’s test.). These findings suggest that theta and beta activity, particularly in BLA, are elicited by social interaction in the presence of allopregnanolone.

### Theta and beta activity in BLA correlated with social interaction

**Figure 3:**
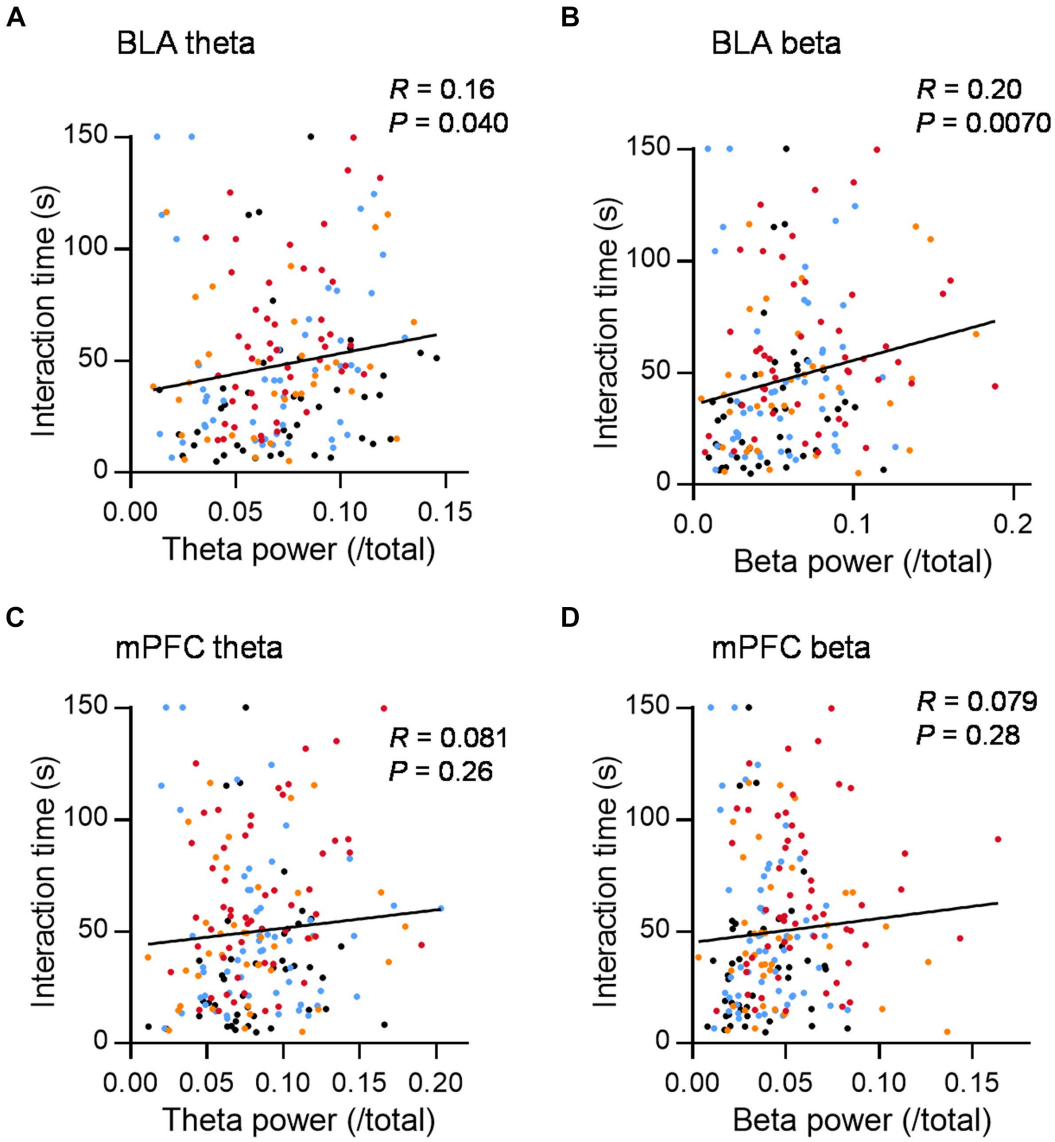
Theta and beta power in the BLA during social interaction are positively correlated with social interaction time. **(A)** The relationship between mean theta power in the BLA during social interaction and social interaction time (150-s bins) in the social interaction test after administration of Veh (black dots), Dia (blue dots), Allo_20 (red dots), and Allo_10 (orange dots). *R* = 0.16, *p* = 0.040, *n* = 172, Pearson correlation coefficient. **(B)** Same as **(A)**, but for the beta power in the BLA. *R* = 0.20, *p* = 0.0070, *n* = 172, Pearson correlation coefficient. **(C,D)** Same as **(A,B)**, but for theta and beta power in the mPFC, respectively. *R* = 0.081 and 0.079, *p* = 0.26 and 0.28 for theta and beta power, respectively, *n* = 193, Pearson correlation coefficient.

To further investigate the relationship between theta or beta activity and social behavior, we plotted the LFP power in the social interaction zone against the interaction time in 2.5 min bins. Theta and the beta power in the BLA were positively correlated with social interaction time (Figures 3A,B; *R* = 0.16 and 0.20, *p* = 0.040 and 0.0070, for BLA theta and beta power, respectively, *n* = 172, Pearson correlation coefficient). Similar trends were observed in mPFC, although the relationship was not statistically significant (Figures 3C,D; *R* = 0.081 and 0.079, *p* = 0.26 and 0.28 for mPFC theta and beta power, respectively, *n* = 193 Pearson correlation coefficient). To investigate how theta and beta activity have effects on the increase of social interaction time, we examined the relationship between theta and beta power difference between inside and outside the interaction zone and the mean inter-event interval of each mouse, which is the shortest in Allo_20 group (Figure 1C). There were negative correlations between the mean inter-event interval and theta and beta power in the BLA and theta power in the mPFC, while there was no significant correlation for beta power in the mPFC (Supplementary Figure S4; *R* = −0.54, −0.46, −0.35, and −0.25, *p* < 0.0001, *p* = 0.0004, 0.0089, and 0.063 for BLA theta, BLA beta, mPFC theta, and mPFC beta, respectively. *n* = 56 conditions, Pearson’s correlation coefficient). Thus, increased theta and beta activity in the social interaction zone are associated with the overall social interaction time by reducing intervals between interactions.

### Decrease in theta-band correlated activity between mPFC and BLA related to social interaction

**Figure 4:**
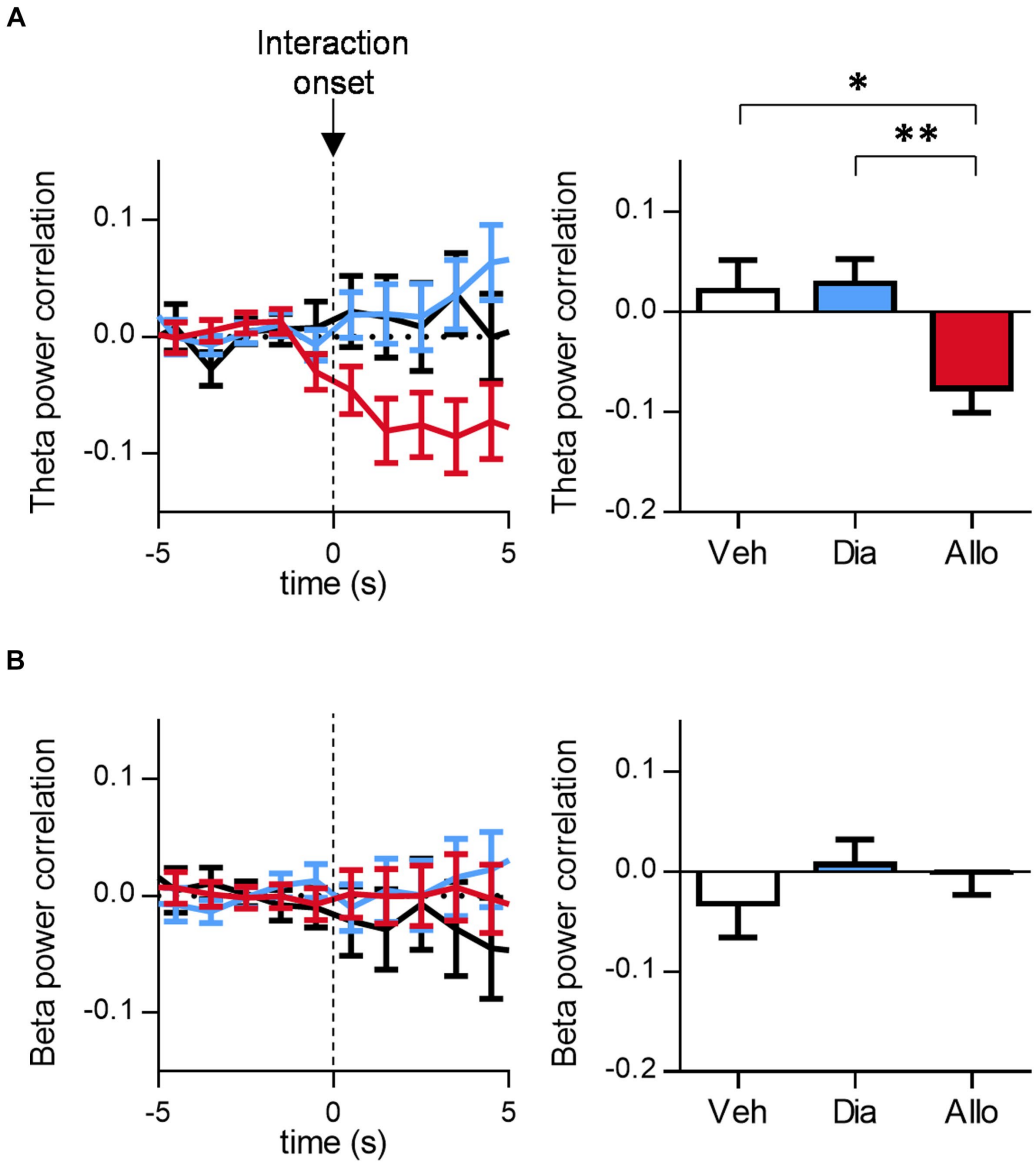
Allopregnanolone, but not diazepam, decreases theta-band correlated activity between mPFC and BLA at the onset of social behavior. **(A)** Time course of mPFC-BLA theta power correlation around the onset of social interaction in administration of vehicle (Veh, black), diazepam (Dia, blue) and allopregnanolone (Allo, red). Baseline was set at the mean of −5~0 s of each event. Right bar graph indicates the mean of 0~5 s after interaction. Data are represented as the mean ± SEM. ^*^p = 0.0382, ^**^p = 0.0059, *n* = 59, 101, and 111 events for Veh, Dia, and Allo, respectively. Tukey’s test. **(B)** Same as **(A)**, but for beta power correlation. *n* = 60, 104, and 113 events for Veh, Dia, and Allo, respectively, Tukey’s test.

Previous studies have reported that activities in mPFC and BLA are involved in processing negative emotions, potentially associated with psychiatric disorders such as PTSD and depression (Norbury et al., 2021). Specifically, theta-band coactivity has been linked to fear and anxiety (Likhtik et al., 2014; Kuga et al., 2022). Therefore, we focused on the correlated activity between mPFC and BLA during the SIT. The mPFC-BLA theta power correlation, compared to baseline, decreased after the initiation of social interactions following the administration of allopregnanolone compared to vehicle and diazepam (Figure 4A; *p* = 0.0382, and 0.0059 for Veh vs. Allo, Dia vs. Allo, respectively. *n* = 59, 191, and 111 events for Veh, Dia, and Allo, respectively. Tukey’s test). Conversely, beta power correlation showed no significant difference across any conditions (Figure 4B; *p* > 0.05, *n* = 50, 77 and 55 events for Veh, Dia and Allo, respectively, Tukey’s test.). Similarly, the mPFC-BLA theta coherence decreased after the initiation of social interactions in mice treated with allopregnanolone, as compared to those treated with the vehicle (Supplementary Figure S5A; *p* = 0.0243 for Veh vs. Allo, *n* = 50, 77and 55 events for Veh, Dia and Allo, respectively. Tukey’s test). The mPFC-BLA beta coherence showed no significant difference between vehicle and allopregnanolone groups, while it increased in Dia compared to Veh (Supplementary Figure S5B; *p* = 0.020 for Veh vs. Dia, *n* = 50, 77and 55 events for Veh, Dia and Allo, respectively. Tukey’s test). These findings indicate that a decrease in theta correlated activity is associated with social interaction following the administration of allopregnanolone, suggesting that the decrease in correlated theta-band activity between mPFC and BLA is involved in social interaction, one of the antidepressant-like behavior.

## Discussion

### Antidepressant**-**like effect of allopregnanolone by increasing frequency of social interaction

In the present study, 20 mg/kg of allopregnanolone exhibited an antidepressant-like effect in the SIT using SDS model mice, whereas diazepam and 10/mg/kg of allopregnanolone did not. When analyzing each social interaction event, we observed that the inter-event interval was shorter in Allo_20 trials than in Veh and Dia trials, even though the duration of each event did not differ among those conditions. These results suggest that allopregnanolone elicits an antidepressant-like effect by increasing the frequency of interaction, rather than the duration. Previous studies have demonstrated that antidepressant drugs such as fluoxetine and ketamine increase the social interaction time in the SIT (Gould et al., 2011; Abe et al., 2019) and our previous data also showed similar results using escitalopram and allopregnanolone (Takasu et al., 2024). However, there have been few studies that quantified the frequency and duration of social interaction events after the administration of antidepressant drugs. In this study, we found that the social interaction time increased due to the frequency of approaches, not the duration. While it is conceivable that allopregnanolone-induced restoration of locomotor activity may have influenced social interaction time, we found no significant correlation between the distance traveled in each trial and the interaction time (Supplementary Figure S3). Furthermore, our previous research has shown that 20 mg/kg of allopregnanolone does not alter the locomotor activity in the open field test (Takasu et al., 2024). These suggest that mice treated with allopregnanolone actively engaged in social interactions rather than merely traversing the interaction zone, a notion supported by the observation that the mean duration of each interaction event was approximately 5–10 s (Figure 1D). Thus, our data may represent a distinctive mechanism underlying the antidepressant-like effects of neuroactive steroids.

### Increase of BLA theta and beta activity at the onset of social interaction

Our previous study demonstrated that theta and beta activities in BLA and mPFC increased when allopregnanolone was administered to mice in their home cages. Specifically, theta activity could be associated with the antidepressant-like effects of neuroactive steroids because diazepam only increased beta activity (Takasu et al., 2024). In the present study, we further investigated whether this concept could be extended to the neural dynamics associated with antidepression-like behaviors such as social interaction. We observed that theta and beta activity in BLA increased following the onset of social interaction after administration of allopregnanolone, whereas there were no such increases with the vehicle or diazepam. The idea that theta activity in BLA is linked to antidepressant-like effects aligns with previous studies (Antonoudiou et al., 2022; Takasu et al., 2024).

On the other hand, the result showing an increase in theta activity only with allopregnanolone, while theta activity did not increase with diazepam or vehicle despite mice administered with these drugs also engaging in social interaction, may appear surprising. One possibility is that theta activity induced by social interaction following the administration of allopregnanolone has an impact on the motivation for following interactions. This could contribute to the shorter inter-event intervals in the Allo_20 condition compared to other conditions. Furthermore, this increase in theta activity was observed in BLA, but not in mPFC. The positive correlation between theta and beta power and social interaction time was able observed solely in BLA. These findings suggest that BLA may play a central role in regulating antidepressant-like behavior.

Recent findings suggest that theta activity can be induced through volume conduction from other brain regions (Lalla et al., 2017) or coordinated by respiration (Karalis et al., 2016; Bagur et al., 2021; Karalis and Sirota, 2022). Although our study did not fully address the origin of theta activity, it is possible that the BLA or the network activity between the BLA and mPFC is, at least partially, the origin of theta waves. Referring previous studies, the optogenetic stimulation by theta waves in the BLA has been shown to have an antidepressant-like effect in the tail suspension test (Antonoudiou et al., 2022). Additionally, theta-band synchronization has been observed between the amygdala and mPFC related with negative emotion (Narayanan et al., 2011), suggesting the importance of theta-coordinated activity between these regions. Together with our results, these studies suggest the potential involvement of BLA theta activity in the control of social behavior in depression-model mice.

### Coordinated activity between mPFC and BLA associated with social interaction

Dysregulation of neural network activity in brain regions is associated with psychological disorders, including MDD. For instance, altered neuronal activity between the amygdala and its related regions, such as mPFC, has been linked to stress vulnerability and negative emotions in MDD patients (Drevets, 2000; Phillips et al., 2003; Deligiannidis et al., 2013; Chase et al., 2014; Deligiannidis et al., 2019). The present study demonstrated that theta coherence between mPFC and BLA decreased after the initiation of social interaction in mice treated with allopregnanolone. This result aligns with a previous study showing theta coherence between dorsomedial PFC (dmPFC) and BLA decreased while non-stressed mice were approaching a target mouse in the SIT (Kuga et al., 2022). Another study reported that theta coherence between these regions increased when mice were exposed to conditioning stimuli paired with an electric shock (CS+) compared to unpaired stimuli (CS−; Likhtik et al., 2014). In human studies, theta coherence increased in response to CS+ compared to CS-during fear learning (Chen et al., 2021). Similarly, the correlation in theta power between these regions was associated with anxiety-like behavior in the open field test (Likhtik et al., 2014). Based on these previous studies, it is conceivable that mPFC and BLA may communicate through theta oscillations during process involving negative emotion, such as fear and anxiety. When combined alongside our results, the reduced communication between mPFC and BLA through theta oscillations may be implicated in the attenuation of rumination on negative information related to stress. Nevertheless, in the vehicle condition, theta power correlation did not show a complete increase at the onset of social interaction (Figure 4A), whereas theta power decrease (Figure 2A) and theta coherence increase (Supplementary Figure S5A) were observed, which is consistent with previous studies (Likhtik et al., 2014; Antonoudiou et al., 2022). One possible factor contributing to the lack of a significant increase in theta power correlation in the vehicle condition may be the difference in aversiveness of the stimuli [a novel ICR mouse in our study or electric stimuli in fear conditioning (Chen et al., 2021)] or the difference in pathological state [SDS mice or non-stressed mice (Kuga et al., 2022)]. It is anticipated that future research will shed light on this aspect.

Neuroactive steroids regulate emotional cognition by altering the functional connectivity between amygdala and frontal cortex (Sripada et al., 2014) and counteract the network activity dysregulated by chronic stress (Antonoudiou et al., 2022). These effects may lead to antidepressant effects of neuroactive steroids, as opposed to benzodiazepines. In this study, we investigated the precise temporal dynamics of neural activities associated with antidepressant-like effects using the SIT. We discovered that theta oscillation in the BLA, along with its coordinated activity with the mPFC induced by allopregnanolone, was associated with social behavior. Therefore, the modulation of theta-band coordinated activities through neuroactive steroids may offer a potential therapeutic target for the treatment of MDD.

## Author’s note

Allopregnanolone but not diazepam elicits antidepressant-like effects through increasing social interaction events and increase theta oscillation in BLA, which is correlated with the onset of social behavior.

## Data availability statement

The datasets presented in this study can be found in online repositories. The names of the repository/repositories and accession number(s) can be found in the article/Supplementary material.

## Ethics statement

The animal studies were approved by the Animal Care and Use Committee of Shionogi Research Laboratories, Osaka, Japan. Electrophysiological assessments were performed according to the AAALAC International guidelines. The studies were conducted in accordance with the local legislation and institutional requirements. Written informed consent was obtained from the owners for the participation of their animals in this study.

## Author contributions

YY: Conceptualization, Data curation, Formal analysis, Investigation, Methodology, Validation, Visualization, Writing – original draft, Writing – review & editing. RT: Data curation, Writing – review & editing. HA: Data curation, Writing – review & editing. SS: Data curation, Writing – review & editing. TO: Project administration, Writing – review & editing. TT: Project administration, Writing – review & editing. KT: Supervision, Validation, Writing – review & editing. KO: Supervision, Validation, Writing – review & editing.

## Supplementary material

SUPPLEMENTARY FIGURE S1 Histological verification of the placement of the tips of electrodes. **(A)** Brain maps for the identified locations (open circles) of the tips of electrodes for the mPFC. *n* = 18 mice. (B) Representative macrograph of the mPFC. The arrowhead indicates the location of the electrode tip. The nuclei were counterstained with DAPI. **(C,D)** Same as **(A,B)**, but for the BLA. *n* = 16 mice.

SUPPLEMENTARY FIGURE S2 Social interaction and locomotor activity decreased after chronic social defeat stress. **(A)** Social interaction time before SDS (dSIT1) and after SDS (dSIT2 and dSIT3). We selected mice which showed lower interaction time in both dSIT2 and dSIT3 than in dSIT1 for implantation of the electrodes for LFP recording. ^****^*P* < 0.0001, *n* = 18 mice, Tukey’s test. **(B)** Same as **(A)**, but for the distance traveled during the SIT. ^****^*P* < 0.0001, *n* = 18 mice, Tukey’s test.

SUPPLEMENTARY FIGURE S3 The relationship between locomotor activity and social behavior. **(A)** Distance traveled during the SIT after administration of vehicle (Veh white column), diazepam (Dia, blue column), allopregnanolone 20 mg/kg (Allo_20, red column), and allopregnanolone 10 mg/kg (Allo_10, orange column). Data are represented as the mean± SEM. *^*^P* = 0.037, *n* = 18, 18, 18, 11 mice for Veh, Dia, Allo_20, and Allo_10, respectively, Tukey’s test. **(B)** The relationship between the social interaction time (150-s bins) and the distance traveled (150-s bins) after administration of Veh (black dots), Dia (blue dots), Allo_20 (red dots), and Allo_10 (orange dots). *R* = 0.023, 0.081, 0.27, and -0.25, *P* = 0.93, 0.75, 0.28, and 0.46, *n* = 18, 18, 18, and 11 mice for Veh, Dia, Allo_20, and Allo_10, respectively. Pearson correlation coefficient.

SUPPLEMENTARY FIGURE S4 Inter-event intervals are negatively correlated with theta and beta power difference in the BLA. **(A)** The relationship between the mean inter-event interval of each mouse and BLA theta power difference between inside and outside the interaction zone. Veh, Dia, Allo_20, and Allo_10 are represented by black, blue, red and orange dots, respectively. Data from mice with three or more events were used to calculate the mean of individuals, *R* = −0.54, *P* < 0.0001, *n* = 56 conditions. Pearson correlation coefficient. **(B)** Same as **(A)**, but for BLA beta power difference. *R* = −0.46, *P* = 0.0004, *n* = 56 conditions. Pearson correlation coefficient. **(C)** Same as **(A)**, but for mPFC theta power difference. *R* = −0.35, *P* = 0.0089, *n* = 56 conditions. Pearson correlation coefficient. **(D)** Same as **(A)**, but for mPFC beta power difference. *R* = −0.25, *P* = 0.063, *n* = 56 conditions. Pearson correlation coefficient.

SUPPLEMENTARY FIGURE S5 Allopregnanolone, but not diazepam, decreases theta coherence between mPFC and BLA at the onset of social behavior. **(A)** Time course of theta coherence between the mPFC and the BLA around the onset of social interaction in administration of vehicle (Veh, black), diazepam (Dia, blue) and allopregnanolone (Allo, red). Baseline was set at the mean of −10~5 s of each event. Right bar graph indicates the mean of 0~5 s after interaction. Data are represented as the mean± SEM. ^*^*P* = 0.0243, *n* = 50, 77 and 55 events for Veh, Dia and Allo, respectively. Tukey’s test. **(B)** Same as **(A)**, but for beta coherence. ^*^*P* = 0.020, *n* = 50, 77, and 55 events for Veh, Dia, and Allo, respectively, Tukey’s test.
