# Supplementary figures and images for: Differential effects of allopregnanolone and diazepam on social behavior through modulation of neural oscillation dynamics in basolateral amygdala and medial prefrontal cortex
Source: Front Cell Neurosci. 2024 Jun 5;18:1404603. doi: 10.3389/fncel.2024.1404603 (PMC11185934; doi:10.3389/fncel.2024.1404603)

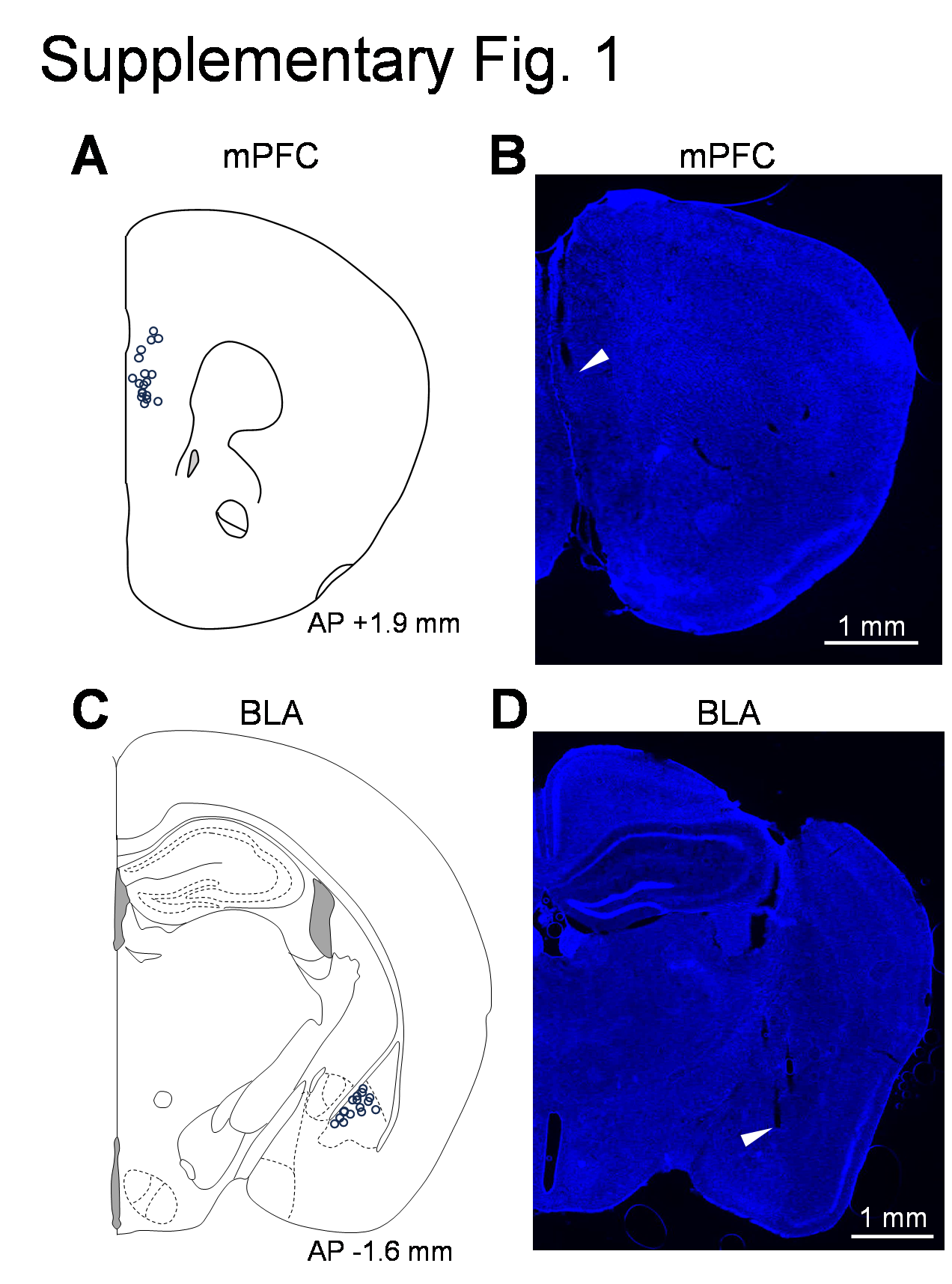

Supplement: SUPPLEMENTARY FIGURE S1 — Histological verification of the placement of the tips of electrodes. (A) Brain maps for the identified locations (open circles) of the tips of electrodes for the mPFC. n = 18 mice. (B) Representative macrograph of the mPFC. The arrowhead indicates the location of the electrode tip. The nuclei were counterstained with DAPI. (C,D) Same as (A,B), but for the BLA. n = 16 mice. [file Image_1.TIF]

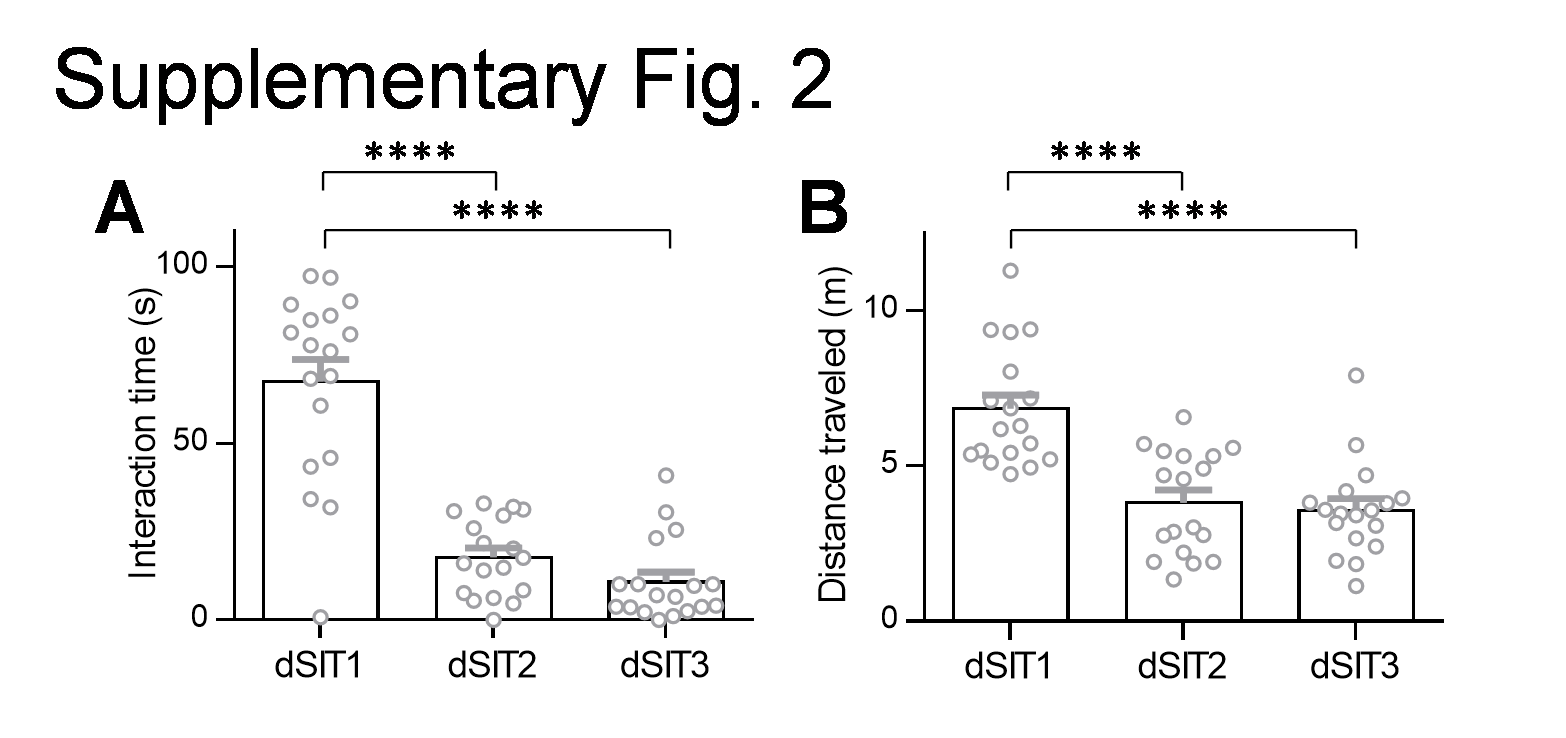

Supplement: SUPPLEMENTARY FIGURE S2 — Social interaction and locomotor activity decreased after chronic social defeat stress. (A) Social interaction time before SDS (dSIT1) and after SDS (dSIT2 and dSIT3). We selected mice which showed lower interaction time in both dSIT2 and dSIT3 than in dSIT1 for implantation of the electrodes for LFP recording. ****P < 0.0001, n = 18 mice, Tukey’s test. (B) Same as (A), but for the distance traveled during the SIT. ****P < 0.0001, n = 18 mice, Tukey’s test. [file Image_2.TIF]

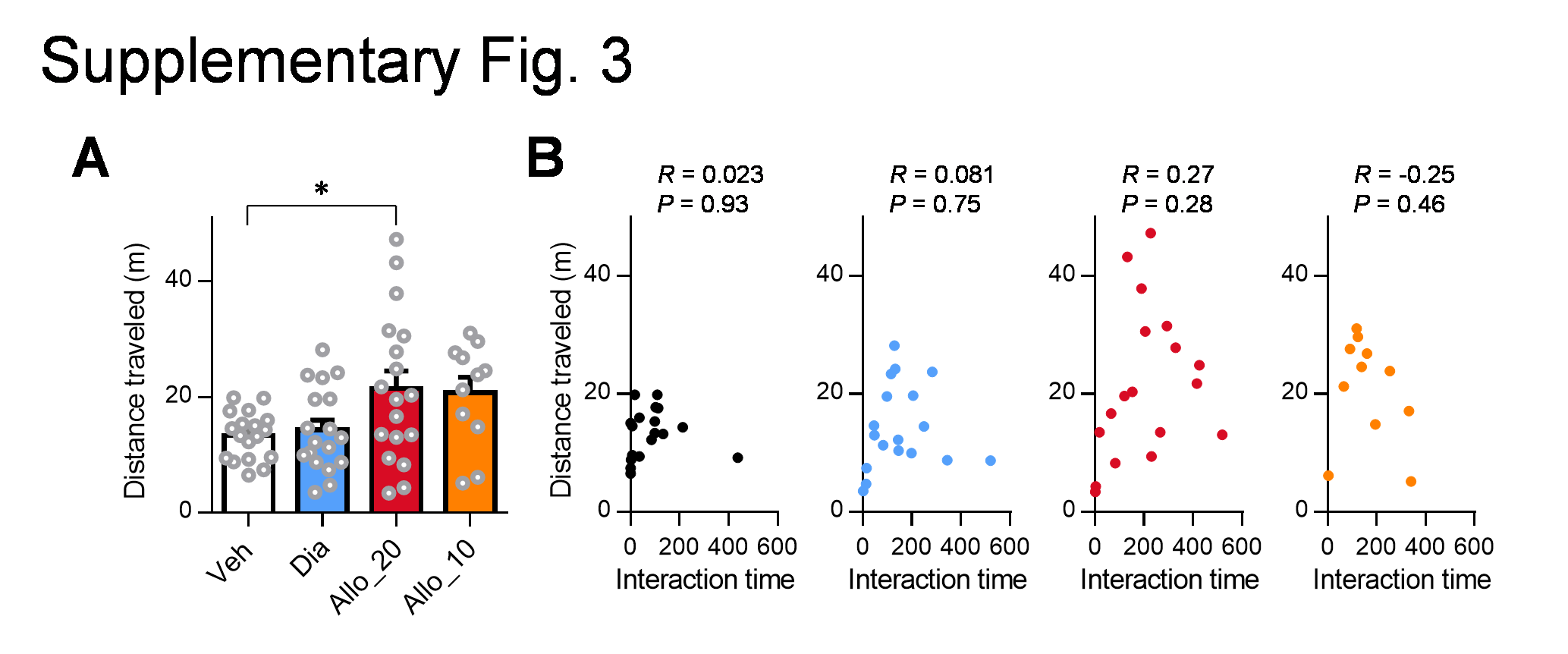

Supplement: SUPPLEMENTARY FIGURE S3 — The relationship between locomotor activity and social behavior. (A) Distance traveled during the SIT after administration of vehicle (Veh white column), diazepam (Dia, blue column), allopregnanolone 20 mg/kg (Allo_20, red column), and allopregnanolone 10 mg/kg (Allo_10, orange column). Data are represented as the mean± SEM. *P = 0.037, n = 18, 18, 18, 11 mice for Veh, Dia, Allo_20, and Allo_10, respectively, Tukey’s test. (B) The relationship between the social interaction time (150-s bins) and the distance traveled (150-s bins) after administration of Veh (black dots), Dia (blue dots), Allo_20 (red dots), and Allo_10 (orange dots). R = 0.023, 0.081, 0.27, and -0.25, P = 0.93, 0.75, 0.28, and 0.46, n = 18, 18, 18, and 11 mice for Veh, Dia, Allo_20, and Allo_10, respectively. Pearson correlation coefficient. [file Image_3.TIF]

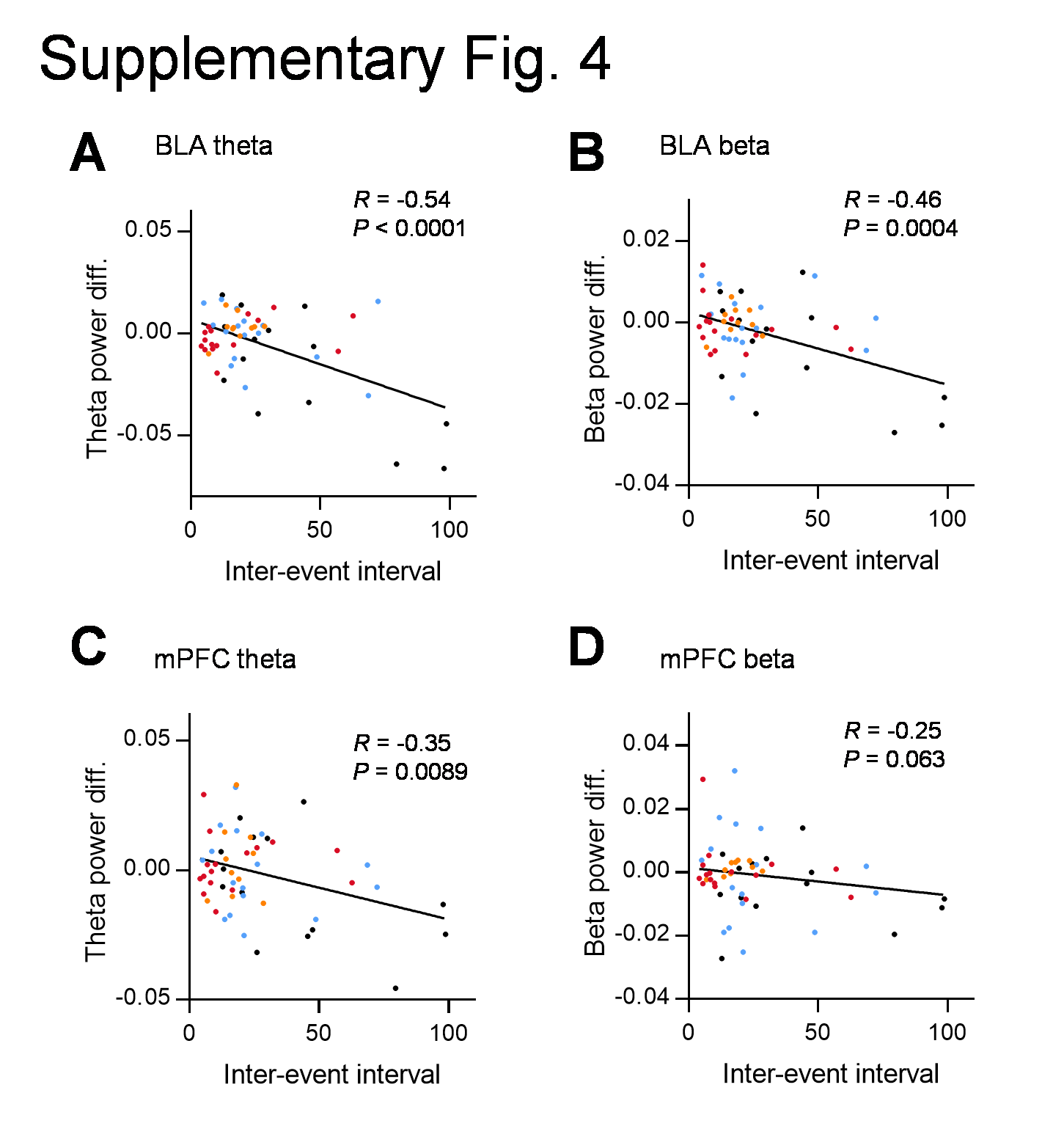

Supplement: SUPPLEMENTARY FIGURE S4 — Inter-event intervals are negatively correlated with theta and beta power difference in the BLA. (A) The relationship between the mean inter-event interval of each mouse and BLA theta power difference between inside and outside the interaction zone. Veh, Dia, Allo_20, and Allo_10 are represented by black, blue, red and orange dots, respectively. Data from mice with three or more events were used to calculate the mean of individuals, R = −0.54, P < 0.0001, n = 56 conditions. Pearson correlation coefficient. (B) Same as (A), but for BLA beta power difference. R = −0.46, P = 0.0004, n = 56 conditions. Pearson correlation coefficient. (C) Same as (A), but for mPFC theta power difference. R = −0.35, P = 0.0089, n = 56 conditions. Pearson correlation coefficient. (D) Same as (A), but for mPFC beta power difference. R = −0.25, P = 0.063, n = 56 conditions. Pearson correlation coefficient. [file Image_4.TIF]

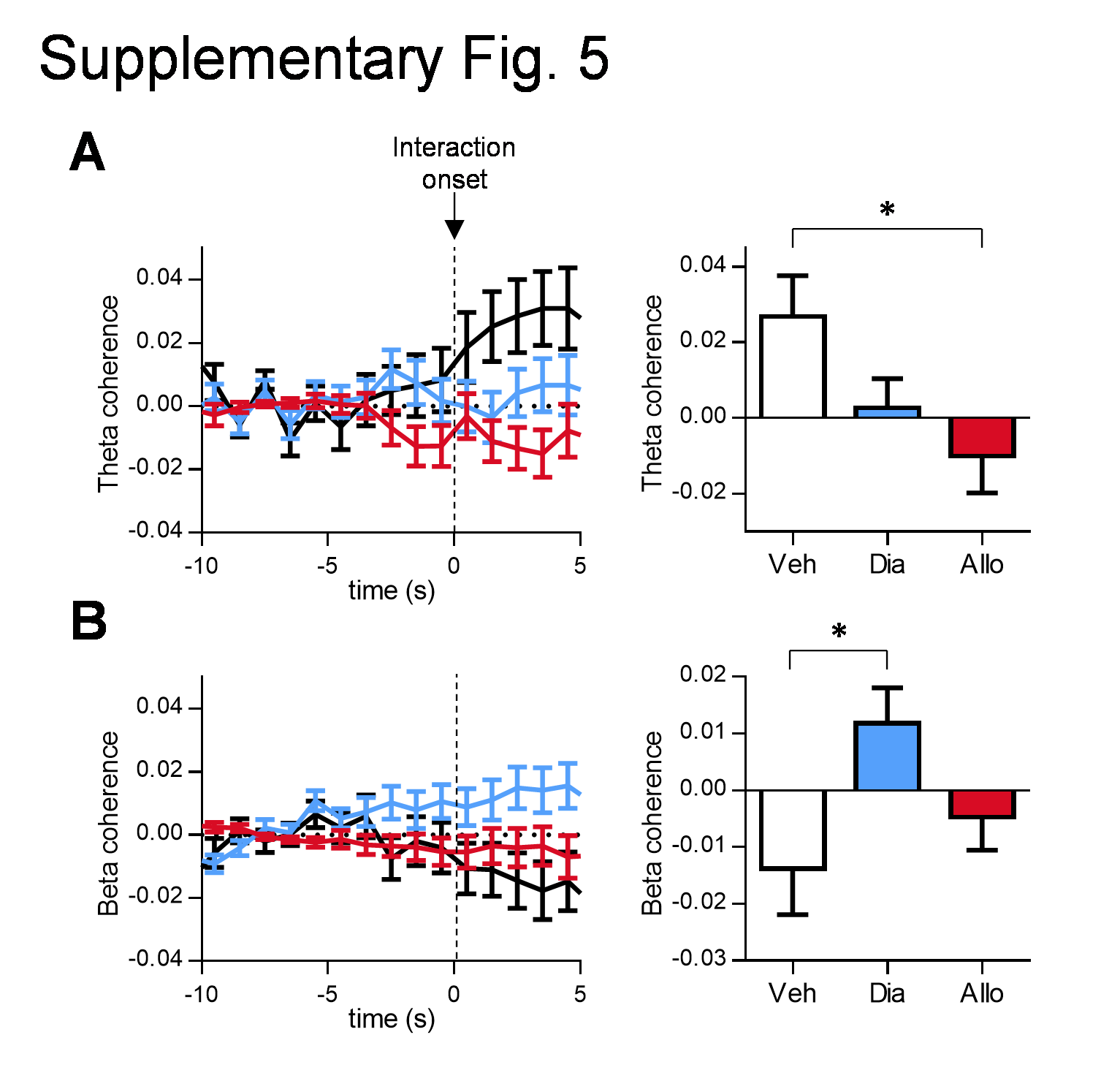

Supplement: SUPPLEMENTARY FIGURE S5 — Allopregnanolone, but not diazepam, decreases theta coherence between mPFC and BLA at the onset of social behavior. (A) Time course of theta coherence between the mPFC and the BLA around the onset of social interaction in administration of vehicle (Veh, black), diazepam (Dia, blue) and allopregnanolone (Allo, red). Baseline was set at the mean of −10~5 s of each event. Right bar graph indicates the mean of 0~5 s after interaction. Data are represented as the mean± SEM. *P = 0.0243, n = 50, 77 and 55 events for Veh, Dia and Allo, respectively. Tukey’s test. (B) Same as (A), but for beta coherence. *P = 0.020, n = 50, 77, and 55 events for Veh, Dia, and Allo, respectively, Tukey’s test. [file Image_5.TIF]
